# Absence of specific autoantibodies in patients with narcolepsy type 1 as indicated by an unbiased random peptide-displayed phage screening

**DOI:** 10.1371/journal.pone.0297625

**Published:** 2024-03-05

**Authors:** Thi-Tuyet Trinh Tran, Thi-Hong Nhung Nguyen, Yves Dauvilliers, Roland Liblau, Xuan-Hung Nguyen

**Affiliations:** 1 Department of Biobank, Hi-Tech Center and Vinmec-VinUni Institute of Immunology, Vinmec Healthcare system, Hanoi, Vietnam; 2 Department of Neurology, Sleep-Wake Disorder Center, CHU Montpellier, Montpellier, France; 3 Department of Inflammatory Diseases of the Central Nervous System: Mechanisms and Therapies, Toulouse Institute for Infection and Inflammatory Diseases, University of Toulouse, Toulouse, France; 4 College of Health Sciences, VinUnivesity, Hanoi, Vietnam; Peking University People’s Hospital, CHINA

## Abstract

Narcolepsy type 1 (NT1) is an enigmatic sleep disorder characterized by the selective loss of neurons producing orexin (also named hypocretin) in the lateral hypothalamus. Although NT1 is believed to be an autoimmune disease, the orexinergic neuron-specific antigens targeted by the pathogenic immune response remain elusive. In this study, we evaluated the differential binding capacity of various peptides to serum immunoglobin G from patients with NT1 and other hypersomnolence complaints (OHCs). These peptides were selected using an unbiased phage display technology or based on their significant presence in the serum of NT1 patients as identified from previous studies. Although the subtractive biopanning strategy successfully enriched phage clones with high reactivity against NT1 serum IgG, the 101 randomly selected individual phage clones could not differentiate the sera from NT1 and OHC. Compared to the OHC control group, serum from several NT1 patients exhibited increased reactivity to the 12-mer peptides derived from TRBV7, BCL-6, NRXN1, RXRG, HCRT, and RTN4 proteins, although not statistically significant. Collectively, employing both unbiased and targeted methodologies, we were unable to detect the presence of specific autoantibodies in our NT1 patient cohort. This further supports the hypothesis that the autoimmune response in NT1 patients likely stems primarily from T cell-mediated immunity rather than humoral immunity.

## Introduction

Narcolepsy type 1 (NT1) is a chronic sleep disorder defined by excessive daytime sleepiness and cataplexy [1,2]. NT1 is attributed to a marked and selective loss of orexin-producing neurons in the lateral hypothalamus [3,4]. Diagnosis typically occurs several years after the onset of clinical symptoms and relies on an invasive procedure measuring the orexin level in the cerebral spinal fluid (CSF) [5–7]. Although assessing the sleep cycle can aid in diagnosing NT1, it may sometimes be confounded with other sleep disorders. Attempts to identify NT1-specific biomarkers, including measuring the cytokines in the CSF of patients, have not yielded distinguishing differences compared to controls [8–10]. Although over 98% of NT1 patients carry the HLA-DQB1*06:02 allele [11], resulting in a 251-fold increase in narcolepsy risk [12], this allele cannot serve as a specific diagnostic target due to its presence in the normal population. Therefore, it is essential to develop a less invasive and specific early-stage diagnostic test for NT1.

The precise mechanism underlying narcolepsy remains unclear; however, a myriad of indirect evidence strongly supports the involvement of autoimmunity in triggering NT1. These include the selective loss of orexin neurons, as aforementioned; the association with polymorphism in immune function-related genes [13,14] and the association between NT1 and a specific H1N1 Pandemrix® vaccine adjuvant [10,15–17]. Recently, additional evidence has emerged further bolstering the autoimmune hypothesis, specifically through identifying autoreactive CD4^+^ and CD8^+^ T cell populations in the serum of the patients compared to HLA-DQB1*06:*02*-positive healthy controls [18,19].

Notably, findings from various studies have hinted at the presence of autoreactive antibodies in patient’s serum, but the data remains unvalidated by other research, and the pathogenicity of the suggested autoantibodies has yet to be elucidated. Anti-NEI/αMSH and anti-HCRTR2 antibodies were suggested as potential target autoantigens, but they were also detected in the sera of patients with other diseases [20,21]. Additional evidence contradicted the hypothesis that anti-HCRTR2 antibodies are autoantibodies triggering NT1 [22,23]. Furthermore, a study attempted to validate nine previously proposed autoantigens found no significant difference in the level of antibodies compared to control relatives [24]. Overall, the exact target autoantigens and autoantibodies responsible for triggering the NT1 remain to be identified.

The present study aimed to identify potential biomarkers that are associated explicitly with NT1. We screened a phage-displayed random peptide library against circulating IgG antibodies purified from NT1 and other hypersomnolence complaint (OHC) patients to identify peptides capable of effectively distinguishing between NT1 and OHC.

## Material and methods

### Ethical approval

The consecutive adult patients with NT1 and other hypersomnolence complaints (OHC) were included at the National Reference Center for Narcolepsy, Montpellier, France between July 2014, and March 2017. This study was approved by the local ethics committees (Committee of Constitution of a cohort and a clinical, neurophysiological, and biological bank of rare hypersomnolence disorders, Narcobank AOM07-138 and The Institutional ethical review board for Biomedical Research, Vinmec International General Hospital JSC). All patients provided written informed consent to participate in the study from the ethics committees by the principles outlined in the Declaration of Helsinki.

### Patient samples

Serum samples collected from well-characterized NT1 patients with recent onset of less than five years following the first symptoms, low CSF orexin-A levels (i.e., <110 pg/mL), and carrying the HLA-DQB1*06:*02* allele were included in this study. All NT1 patients meet international classification of sleep disorders 3 (ICSD3) criteria for type 1 narcolepsy. Tightly age-, sex- and HLA-DQB1*06:*02*-matched patients with other hypersomnolence complaints (OHC) were selected as control subjects for phage display screening (Table 1). All patients with OHC had complaints of excessive daytime sleepiness, with normal polysomnography and multiple sleep latency test values and normal CSF orexin-A levels. These serum samples were collected within five years from the estimated disease onset and represented both genders, generously provided by Prof. Yves Dauvilliers (CHU de Montpellier, INSERM U1061, Montpellier, France).

**Table 1:** Patient demographic for Phage screening.

	Type 1 Narcolepsy						Other hypersomnolence complaints				
	Sample ID	Gender	Age	BMI	EDS onset	HCRT pg/l	Sample ID	Gender	Age	BMI	HCRT pg/l
1	ARN	F	33	29	28	10	ACQ	F	34	18.59	308
2	ARM	F	17	22	15	17	CAM	F	16	18.22	243
3	BOU	M	49	23.85	48	29	MAS	M	48	N/A	615
4	CAR	F	22	18.78	20	28	LAU	F	20	17.67	258
5	CRO	M	33	30.35	33	1.4	GIN	M	32	26.87	301
6	DUR	F	26	21.77	23	15	MAS	F	28	20.4	248
7	FAB	F	19	24.61	15	10	LOP	F	18	20.83	287
8	GUI	M	16	20.76	14	86	MAT	M	16	19.02	314
9	MAH	M	16	19.57	11	11	GUE	M	18	20.6	210
10	MIC	F	38	33.71	37	0	HUE	F	40	23.15	453

### Serum IgG purification

The purification of IgGs from pooled or individual serum samples was carried out using AbraMag™ anti-Human IgG Magnetic Beads (Thomas Scientific). The volume of reagents indicated herein is a respective amount to purify each 50 μl serum and can be adjusted accordingly. In short, 100 μL (0.5 mg) of beads was washed twice with 1 mL of binding buffer (0.05% Tween-TBS) using a magnetic stand for 2 minutes. The beads were resuspended in 450 μL of binding buffer, and 50 μL of serum was added to the mixture. After being gently mixed on a rotator for 30 minutes, the IgGs bound to the beads were separated from unbound proteins using the magnetic stand and washed twice with 0.5 mL of binding buffer. Elution of the IgGs from the beads was achieved by incubating them with 100 μL of 0.1 M Glycine pH 2.0 for 10 minutes at room temperature, with occasional gentle mixing. The eluent was removed from beads on the magnetic separator and then neutralized with 15 μL of neutralization buffer (1M Tris pH 8.0).

### Peptide phage display

**Fig 1:**
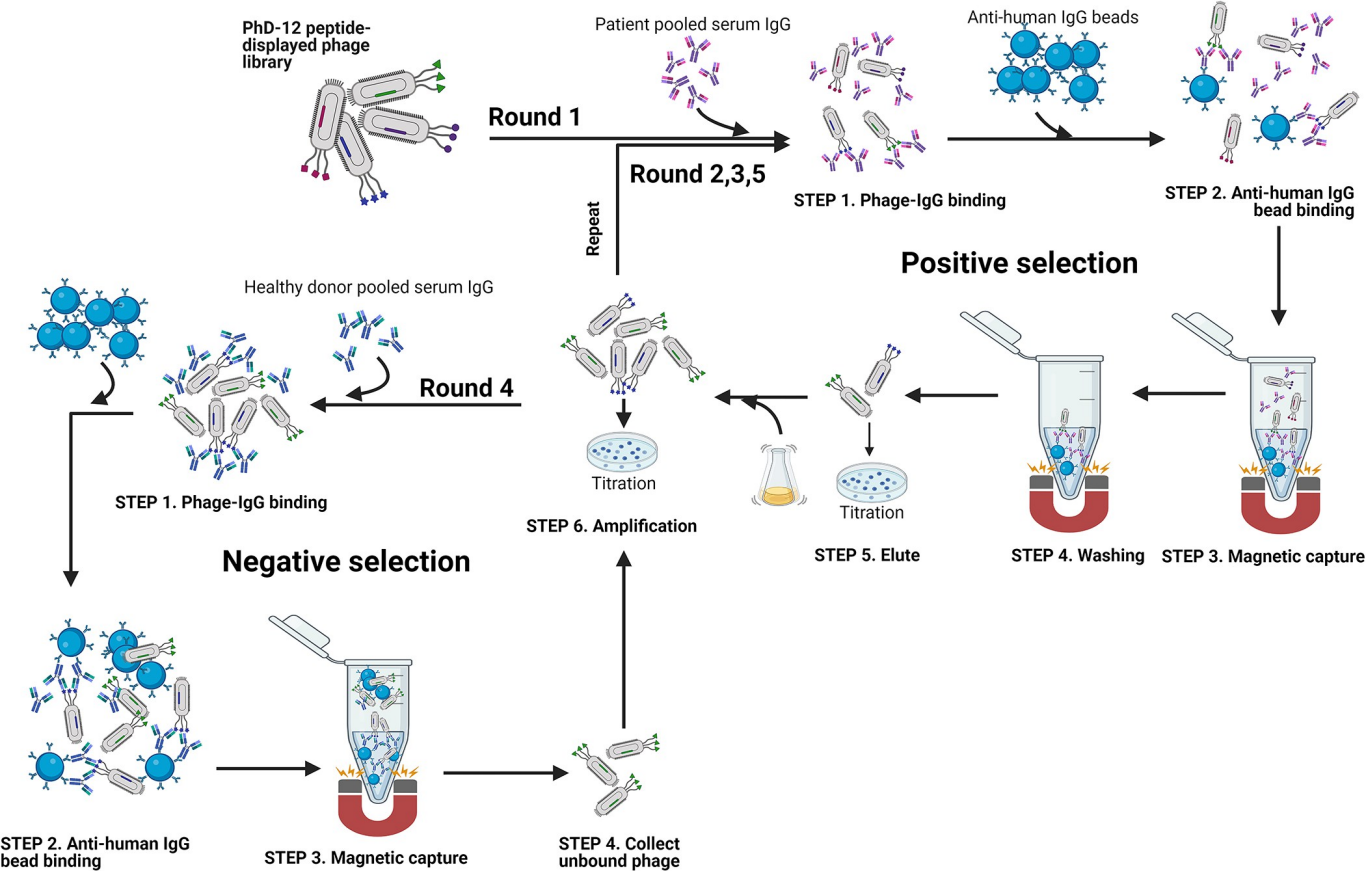
Schematic representation of the phage display panning procedure. The positive selection (the 1st, 2nd, 3^rd^, and 5th rounds) and negative selection (the 4th round) were performed against NT1 and OHC pooled serum IgG, respectively.

For peptide selection, a random dodecamer peptide combinatorial library fused to the pIII capsid of the M13 phage, Ph.D.-12 (NEB) was incubated with pooled IgGs coupled to magnetic beads. Five rounds of biopanning were performed using a subtractive strategy in which the 1^st^, 2^nd^, 3^rd,^ and 5^th^ rounds were positive biopanning against pooled IgGs from patients. In contrast, the 4^th^ round, subtractive biopanning, was performed against pooled IgGs from patients with OHC (Fig 1). In each round of positive biopanning, a diluted phage library including approximately 3x10^10^ pfu/mL was incubated with IgGs from pooled NT1 serum to a final volume of 200 μL with TBS = 0.1% Tween (TBST) for 20 minutes at room temperature (RT). The phage-IgG mixture was then added to anti-human IgG beads pre-blocked for 1 hour with TBST. After 15 minutes of incubation at RT with occasional mixing, magnetic separation, and 10-time washing with TBST, unbound phages were removed. The phages bound to the magnetic beads were eluted with acidic elution buffer (0.1 M Gly-HCl, pH 2.2) and then immediately neutralized with 1 M Tris-HCl, pH 9.1. The neutralized phages were propagated in *Escherichia coli* ER2738 (NEB) and used as input for the subsequent positive biopanning round. For the 4^th^ round of subtractive biopanning, the amplified phage library from the 3^rd^ round was incubated with IgGs purified from pooled serum of 10 tightly age-, sex- and HLA-DQB1*06:*02*-matched patients with other hypersomnolence complaints (OHC), and unbound phases were collected using magnetic separation. They were then propagated and used as input in the last round of positive selection.

### Phage binding ELISA

Following the fifth round of biopanning, an enriched phage library against NT1 patient-specific IgGs was spread on the IPTG/X-gal/Tet^+^ agar plates to select the interest clones, which appeared as blue plaques. 101 phage clones were randomly picked for amplification and assessed for their reactivity and binding specificity to pooled sera from patients with NT1 and OHC using indirect ELISA. Phage clones that displayed significantly higher binding capacity to NT1 pooled serum IgGs than to OHC pooled serum IgGs were individually tested with IgGs purified from sera of 10 individual NT1 and 10 matched OHC patients. Briefly, each well of a 96-well flat-bottomed Nunc Maxisorp plate (Thermo Scientific) was coated with at least 10^9^ phage particles diluted in 100 μL of coating buffer (0.15 M Na_2_CO_3,_ 0.035 M NaHCO_3_, pH 9,6) and incubated overnight at 4°C. After removing the coating solution, the wells were washed thrice with PBST (D-PBS containing 0.05% (v/v) Tween-20). Blocking buffer (5% BSA in PBS) was added to each well and incubated for 1.5 hours at room temperature. Pooled OHC- and NT1-derived IgG stocks were diluted 1200 and 1000 times, respectively, in blocking buffer and added to the wells, followed by incubation for one hour at room temperature. The plates were then washed three times with PBST and incubated with 100 μL of horseradish peroxidase (HRP) conjugated goat-anti-human IgG (Abcam, diluted 30000 times in 5%Tween/PBS) for 1 hour at room temperature. Three additional washes with PBST were performed, and each well was added with 100 μL of substrate solution containing 3,3′,5,5′- tetramethylbenzidine (TMB) (Abcam) and incubated for 15 minutes at room temperature to develop the readable color. The color development was halted by adding 100 μL/well of 1 N HCl, and the plates were subsequently read at 450nm. To account for any signal differences arising from variations in IgG concentrations between NT1- and OHC-derived IgGs, wells coated with the coating buffer without phages were included as blank.

### Indirect ELISA

We used the indirect ELISA method to evaluate the affinity of twenty-six NT1 and eight OHC serum (demography described in Supplementary data 1) to 15 selected peptides that have been shown to be abundant in patients with NT1 [25,26]. All peptides used in this study were synthesized by Genscript (USA) and were delivered as crude lyophilizates. The experimental procedures closely resembled the aforementioned phage binding ELISA, with variation: rather than employing phage particles for coating, the plate was coated with target peptides at the concentration of 100 μg per well. After incubating overnight at 4°C, the plate was blocked by PBS 5% BSA for one hour at RT. Antibodies in serum samples were incubated with plates immediately after washing, and the ELISA procedure was performed as described above.

### Statistics

An unpaired t-test with Welch’s correction was used to determine significant differences in reactivity between groups. All statistical analyses were performed using GraphPad Prism 9.5.0 software, and p-values less than 0.05 were considered statistically significant.

## Results

### Enrichment of phage titer after subtractive biopanning strategy

To identify peptides that specifically bind to potential autoantibodies in NT1 patient serum, we screened a phage-displayed random peptide library (Ph.D -12, NEB) against IgGs derived from pooled serum of 10 recent onset NT1 patients (Table 1) using a subtractive biopanning strategy. To subtract phage clones displaying peptides that non-specifically bind to IgG in human serum, we performed negative panning using IgG derived from pooled serum of 10 OHC subjects at round 4. Before panning, we purified IgG from both pooled OHC and NT1 sera to ensure the exclusion of any unexpected binding of displayed peptides with other serum components.

**Fig 2:**
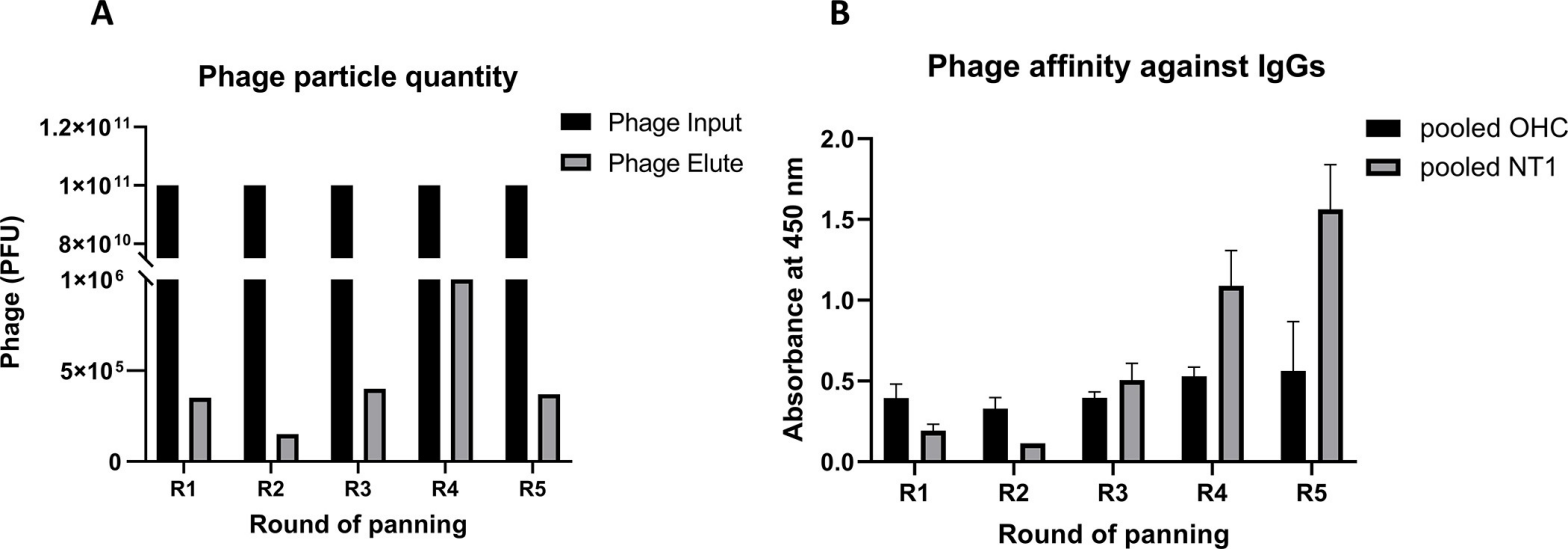
The persistence of phage output after each round of panning. **A.** The white bars indicate the input number of random peptide-displayed phage particles in each round of panning, which is persistently kept at 3x10^9^ PFU. The black bars show phage particle titration at the ends of each round before they were amplified for the next round of input. **B. Increased phage affinity against IgG OHC and NT1 pooled serum after each round of panning.** The binding capacity of enriched phage pools to patient IgGs was measured by phage Elisa. The presenting data are mean ± SD (n = 2).

For each round of panning, the input phage titers were maintained uniformly at approximately 3x10^10^ PFU/mL to ensure the presentation of multiple copies of each specific phage in the library. We monitored the panning efficiency by titrating the eluted phages after each round of panning. Among five rounds of panning, a consistent recovery of about 10^6^ phage PFU from 3 first round of positive panning was observed (Fig 2A). When the phage pool from the third panning was affinity selected against OHC-derived IgG, the phage pool size was significantly reduced, indicating efficient removal of non-specific library members that bound to IgG present in normal serum.

To assess the binding specificity of the libraries obtained from each round of panning to patient’s IgGs, we evaluated their reactivity against pooled serum IgG from OHC and NT1 patients using indirect ELISA. With equal phage input, the binding affinity of the acquired libraries against patientpooled IgGs steadily increased after each round of panning, with a significant surge after the negative panning at round 4 (Fig 2B). These results indicated that the phage panning procedure enriched clones with high reactivity against NT1-pooled serum IgG.

### Binding capacity of randomly selected individual phage clones

**Fig 3:**
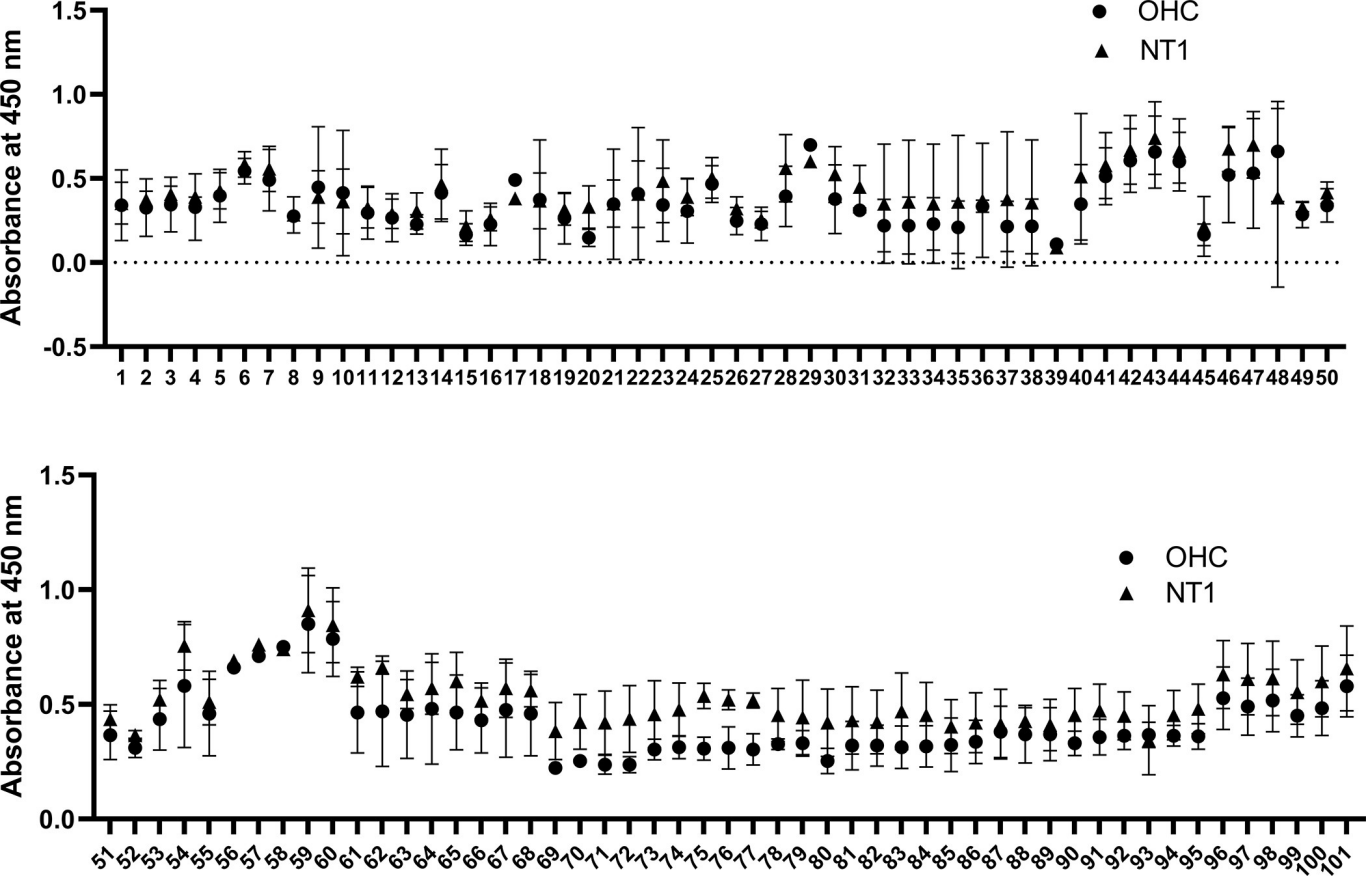
The binding of 101 phages with OHC and NT1 sera. After five rounds of positive and negative biopanning, 101 randomly selected phage clones were evaluated for binding to IgG from pooled OHC and NT1 serum via phage ELISA (n = 3). The binding of phage with OHC pooled serum-derived and NT1 pooled serum-derived IgG are analyzed by multiple unpaired t-tests with p<0.05.

With the final output of phage panning showing significantly increased affinity against NT1 pooled serum IgG, we expected the presence of phage clones displaying peptides that can bind to patient pooled IgG with higher affinity compared to OHC pooled serum IgG. We spread the acquired panning library on IPTG/X-gal/Tet+ agar plates to isolate clones of interest, represented as blue plaques. Randomly, 101 individual phage clones were selected from the plates and amplified to reach at least 10^12^ PFU/mL/clone for further assessment of their affinity to IgGs from pooled OHC and NT1 sera by indirect ELISA (Fig 3).

**Fig 4:**
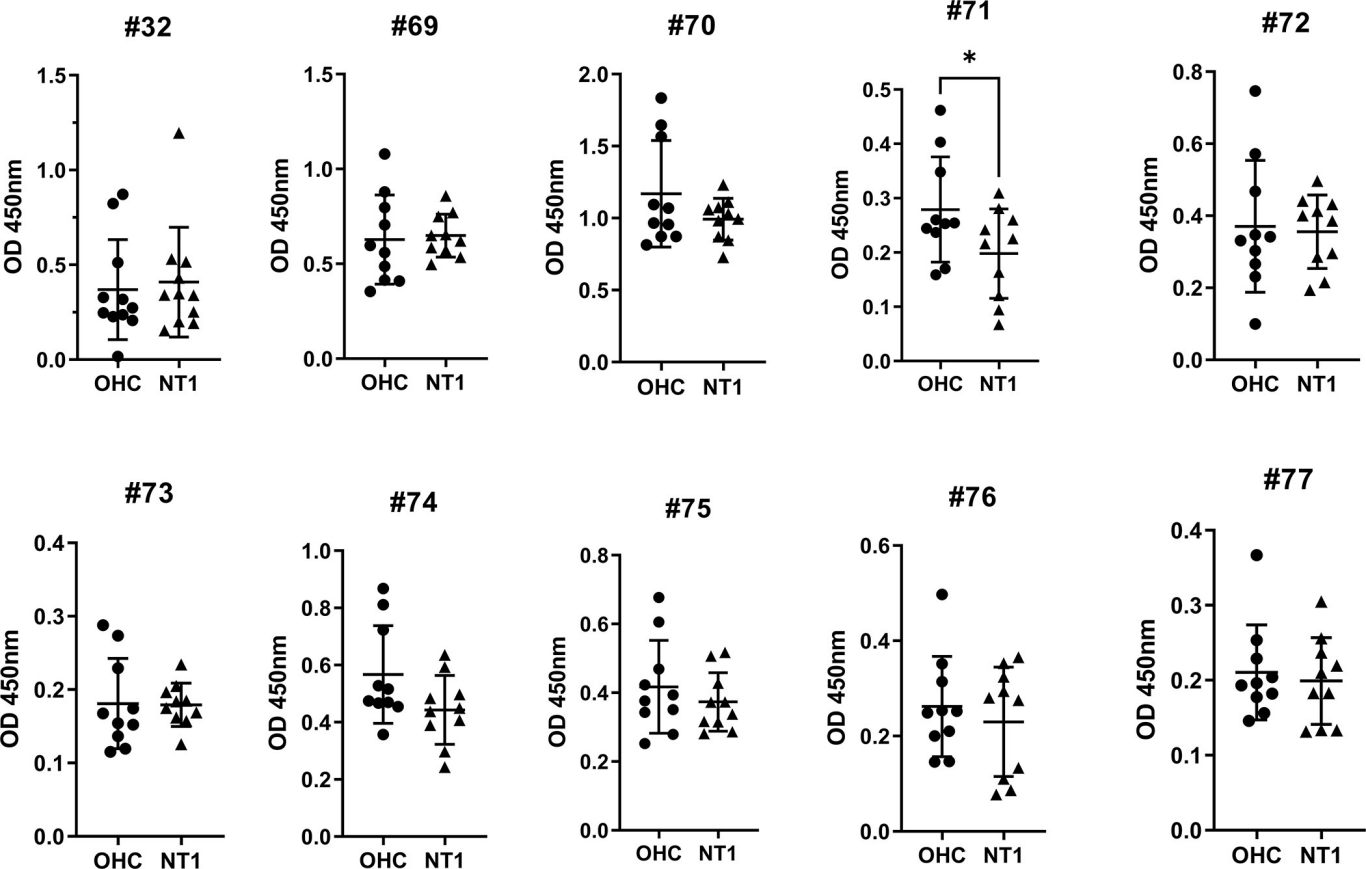
Binding of each phage clone toward individual serum IgG from 10 NT1 and 10 OHC. Chosen phages were validated for specificity by indirect ELISA using individual IgG samples purified from OHC (n = 10) and NT1 serum (n = 10). Error bars indicate mean +/- SD, the paired t-test *p* values has been used to assess statistical significance.

Although no significant difference was observed between the groups, we noted a higher signal pattern of phage clones to the NT1 pooled serum IgG with significant fluctuations among repetitions, as indicated by the extended error bars at each column. Therefore, we selected ten clones (32, 69, 70, 71, 72, 73, 74, 75, 76, and 77) with the most consistent binding potential to IgG from pooled NT1 serum compared to that from pooled OHC serum for further confirmation by ELISA. Upon further examination, we found no significant difference in the mean binding of each selected phage clone to IgGs derived from OHC and NT1 serum except the clone #71 exhibiting higher signals to OHC group (Fig 4). These results suggested that the isolated individual phage clones might not adequately represent the binding capacity of the phage pool after panning.

### The reactivity of NT1 and OHC patient sera to the selected 12-mer peptides

Since the phage display approach failed to isolate the potential peptides specifically bound to IgGs from NT1 patient serum, we selected fifteen 12-mer peptides previously reported to be abundant in patients with NT1 [25,26] and evaluated their affinity to IgG from 26 NT1 and 8 OHC individuals (Supplementary data 1) using ELISA.

Among these 15 peptides (listed in Table 2), the HCRT 54–65 NH_2_ peptide has been reported as an autoreactive antigen in NT1 patients due to increased autoreactivity of CD4^+^ T cells from the patients to this peptide [25]. Another peptide, NRXN1, has been identified through the whole-proteome and targeted design peptide microarrays as a potential autoimmune target in the NT1 [26].

**Table 2:** List of peptide sequences.

	Name	Sequence
1	HCRT 54–65	HGAGNHAAGILT
2	NRXN1	LHTGKSADYVNL
3	SOCS7	SSPGRGGGGGGR
4	BLC6	EGLKPAAPSA
5	CNTN2	RDATKITLAPSS
6	DYSF	FPDPYTELNTGK
7	RXRG	SMSPSAALSTGK
8	SCN5A	AMKKLGSKKPQK
9	ABL2	TQEGGKKAALGA
10	RTN4	MEAPLNSAVPSA
11	SOX11	GGSAGGGAGGAQ
12	TRAV26-1	CIVRAGGTSYGKL
13	TRAV13-1	CAAPGANNLFF
14	TRBV7-9	CASSHSTDTQYF
15	TRAV17	ASAYTGTASKLTF

**Fig 5:**
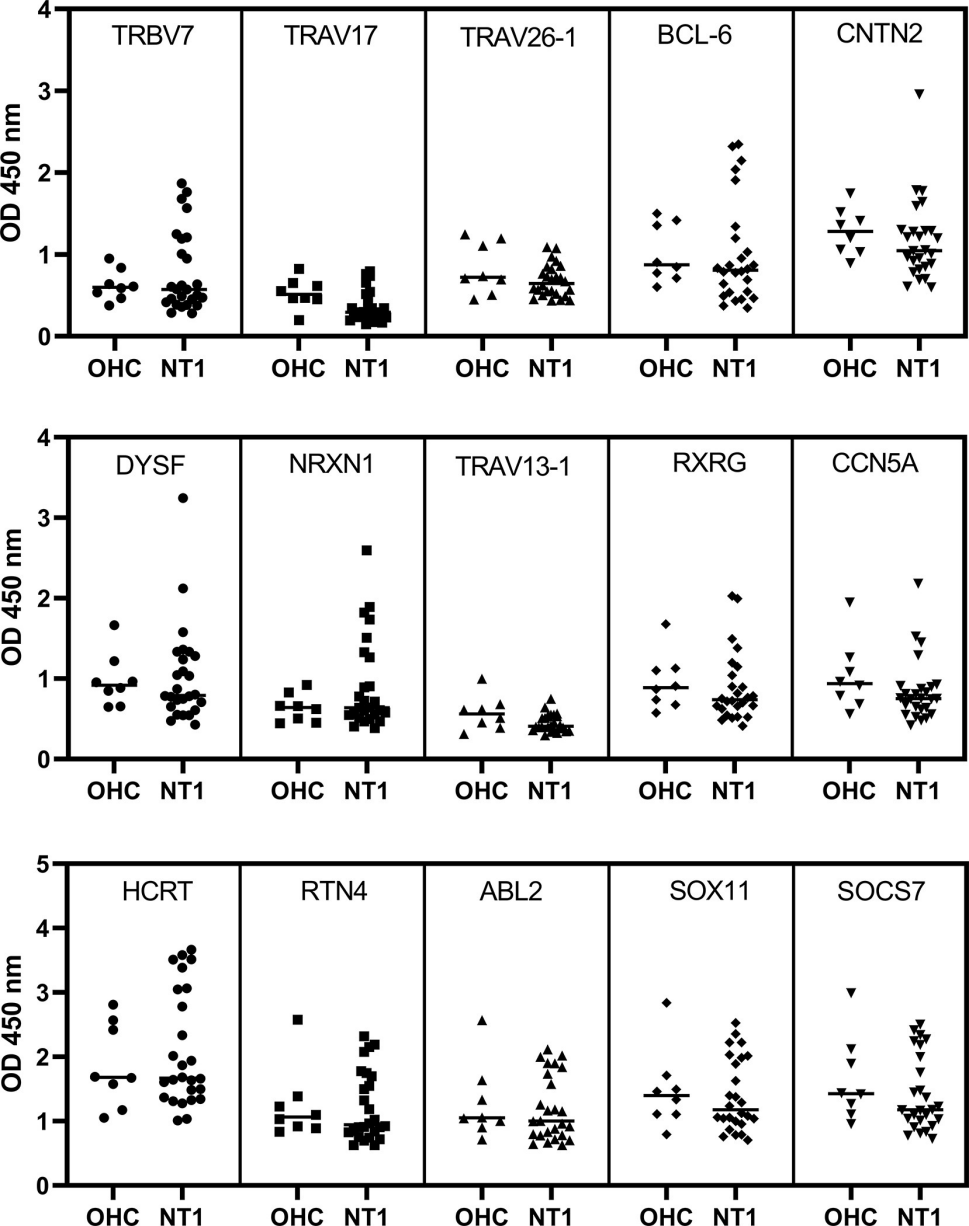
Reactivity of serum antibody against the selected peptides assessed by ELISA. Each dot represents an individual sample from the eight patients with other hypersomnolence complaints (OHC) and twenty-six patients with Narcolepsy type I (NT1). Multiple t-test p values are reported for the group comparison between OHC and NT1.

Interestingly, proteins highly enriched in orexin-producing neurons and involved in synaptic adhesion exhibited significant enrichment in several NT1 patients (Fig 5). We observed an increased reactivity to the peptides derived from TRBV7, BCL-6, NRXN1, RXRG, HCRT, and RTN4 proteins in numerous NT1 patients when compared to the OHC control group. Among these, the hypocretin peptide exhibited the highest signals, although it did not reach statistical significance, likely due to not all NT1 patients showing reactivity to this peptide. On the other hand, the results indicated that samples with high signals for the surveyed peptides were independent of gender, age, BMI, or CSF hypocretin concentration.

## Discussion

NT1 remains a complex disorder, and its formal classification as an autoimmune disease is still under discussion [27]. However, emerging evidence has been accumulating to support the hypothesis of an autoimmune component in the pathogenesis of NT1. Many studies have attempted to identify the target antigens triggering NT1, but so far, with limited success. Our study aimed to identify potential autoantigens triggering NT1 by employing an unbiased, explorative, and data-driven approach.

Recently, we observed elevated levels of B cell-supporting cytokines and impaired follicular T helper cells in the peripheral blood of patients with NT1, suggesting the plausible role of B cells in the disease development [28,29]. Nevertheless, the observations suggesting the existence of autoreactive antibodies in NT1 patients’ serum [30] have not been replicated by independent studies. Therefore, we adopted phage display technology to explore the presence of specific autoantibodies in NT1 patients’ sera, which could indicate the underlying mechanisms driving the disease. Such an approach has successfully identified pathological antigens and etiological agents in several other autoimmune diseases with unknown etiological agents and pathological antigens [31–33]. Through our subtractive biopanning process, we aimed to identify potential peptides from a random peptide-displayed phage library that could exhibit a higher binding to NT1 serum IgG compared to OHC serum IgG.

After three positive and one negative panning, we observed a notable enhancement in the specificity of the acquired phage output against NT1 IgG. With the subtractive panning strategy, we removed the phages that non-specifically bind to OHC pooled IgG (Fig 2A), resulting in a significant rise in the phage specificity against the NT1 IgG (Fig 2B). Subsequently, we randomly selected 101 clones to confirm their IgG binding specificity, of which the ten most promising clones were further validated on serum from individual NT1 and OHC patients. Unexpectedly, none of these candidates demonstrated the ability to differentiate between the serum samples from NT1 and OHC. This observation suggests that the selected individual clones may not sufficiently reflect the serum IgG-binding capacity of the post-panning phage pool. Notably, autoantibodies against orexin-receptor 2 have been detected in NT1 patients [20], but they were also found in many healthy donors, raising questions about their pathogenic relevance. This highlights the complexity of autoantibody profiling in NT1 and suggests that further investigations are needed to elucidate the specific autoantibodies and their roles in NT1 pathogenesis. Furthermore, similar to other multifactorial autoimmune disorders such as type 1 diabetes, multiple autoantibodies may play a role, yet not all are detectable in the majority of patients.

It is essential to consider that the pool of NT1 patient-specific autoantibodies may exhibit significant diversity and be present in minimal quantities in patients’ blood after the disease onset. In fact, the immune response triggered to produce antibodies against a total of approximately 70,000 orexin neurons might not be robust. As a result, the increased affinity of the amplified phage library can be attributed to the collective effect of multiple phage clones binding to various IgGs present in the patients. The onset of NT1 typically occurs within a few weeks after Pandemrix® vaccination [34]. Therefore, the detection of autoreactive IgGs in the total serum of NT1 patients a few years after the disease onset may be challenging. Hence, it may be essential to combine multiple clones for effective differentiation between NT1 and OHC. Alternatively, a phage display approach can be employed to screen for autoantibodies in the cerebrospinal fluid (CSF) rather than the serum of NT1 patients.

Many autoimmune diseases, including type 1 diabetes, systemic lupus erythematosus, Addison’s disease, and Graves’ disease, primarily involve autoantibodies that detect conformational epitopes [35–38]. As discussed recently [39], there is a possibility that autoantibodies in NT1 patient serum also bind to antigens in a conformation-dependent manner that cannot be adapted by short synthetic peptides displayed on our phD-12 phage library.

Since our unbiased phage display approach could not identify the specific peptides associated with NT1, we switched to a targeted strategy using the 12-mer peptides that had previously been demonstrated to be abundant in patients with NT1 [25,26]. The binding capacity of serum IgG from NT1 patients to NRXN1 and HCRT-derived peptides tended to be higher when compared to OHC patients, although these differences did not reach statistical significance. Interestingly, it has been demonstrated in other studies that HCRT and NRXN1 could be the potential autoantigens in NT1 [25]. The absence of potential candidates identified through our peptide library screening for serum IgG binding indirectly strengthens the hypothesis that the autoimmune reaction in NT1 patients predominantly stems from T cell-mediated immunity, as supported by several studies [40–42]. The contribution of autoantibodies to the NT1 pathogenesis may be limited or secondary to T cell-initiated degradation of hypocretin.

In summary, our study sheds light on the complexities surrounding autoantibody-autoantigen interactions that prevent a clear-cut characterization of humoral immunity’s involvement in NT1. However, our unsuccessful initial approach corroborates the finding that autoantigens in NT1 may primarily evoke a cell-mediated autoimmunity response rather than a humoral one. Our study highlights the need for further in-depth investigations, focusing specifically on CD4^+^ and CD8^+^ cells, to unveil the specific autoantigens and their significance in the pathogenesis of NT1.

## Supporting information

S1 Table Patients demography for 12-mer peptide reactivity. (DOCX)

## Abbreviations

CSF: Cerebral spinal fluid
ELISA: Enzyme-linked immunosorbent assay
HLA: Human Leukocyte antigens
iPSC: induced pluripotent stem cell
NT1: Narcolepsy type 1
OHC: other hypersomnolence complaints
Ph.D: Phage Display
