# Supplementary material for: Absence of specific autoantibodies in patients with narcolepsy type 1 as indicated by an unbiased random peptide-displayed phage screening
Source: PLoS One. 2024 Mar 5;19(3):e0297625. doi: 10.1371/journal.pone.0297625 (PMC10914298; doi:10.1371/journal.pone.0297625)
Supplement: S1 Table — (DOCX) [file pone.0297625.s001.docx]

**Supplementary 1: Patient demographic for 12 mer-peptide reactivity assay**

|  | **Type 1 Narcolepsy** | | | | | | **Other hypersomnolence disorders** | | | | |
| --- | --- | --- | --- | --- | --- | --- | --- | --- | --- | --- | --- |
|  | Sample ID | Gender | Age | BMI | EDS onset | HCRT pg/l | Sample ID | Gender | Age | BMI | HCRT pg/l |
| 1 | BAN | M | 35 | 25.5 | 16 | 15 | ASS | F | 50 | 24.97 | 277 |
| 2 | BOU | M | 55 | 22.84 | 17 | 10 | FRA | F | 49 | 33 | 328 |
| 3 | CAG | M | 45 | 30.47 | 44 | 10 | GRA | F | 34 | 28.3 | 250 |
| 4 | CAN | F | 13 | 18.82 | 13 | 32 | JEA | F | 45 | 16.38 | 317 |
| 5 | COS | M | 49 | 28.95 | 36 | 0 | LER | F | 13 | 15.25 | 342 |
| 6 | DEH | M | 32 | 28.41 | 31 | 10 | LUY | F | 23 | N/A | 311 |
| 7 | DEL | F | 45 | 21.8 | 41 | 97 | PAG | F | 35 | N/A | 334 |
| 8 | DIJ | M | 21 | 20.94 | 17 | 10 | SAL | F | 16 | 21.36 | 268 |
| 9 | DUB | M | 35 | 27.68 | 18 | 77 |  |  |  |  |  |
| 10 | FOU | M | 44 | 34.26 | 40 | 51 |  |  |  |  |  |
| 11 | GOE | F | 16 | 19.82 | 10 | 23 |  |  |  |  |  |
| 12 | GUI | M | 32 | 24.16 | 28 | 18 |  |  |  |  |  |
| 13 | HEC | F | 21 | 29 | 16 | 13 |  |  |  |  |  |
| 14 | HEN | F | 30 | 22.04 | 22 | 46 |  |  |  |  |  |
| 15 | HEN | F | 28 | 23.23 | 17 | 41 |  |  |  |  |  |
| 16 | LAJ | F | 17 | 18.73 | 12 | 0 |  |  |  |  |  |
| 17 | MAN | M | 20 | 25.76 | 19 | 16 |  |  |  |  |  |
| 18 | MAR | F | 17 | 23.92 | 11 | 10 |  |  |  |  |  |
| 19 | MAR | M | 48 | 43.42 | 45 | 14 |  |  |  |  |  |
| 20 | NAV | F | 31 | 22.04 | 28 | 20 |  |  |  |  |  |
| 21 | PIO | M | 21 | 24.49 | 18 | 15 |  |  |  |  |  |
| 22 | ROU | M | 54 | 31.22 | 28 | 10 |  |  |  |  |  |
| 23 | SCH | M | 34 | 29.26 | 18 | 25 |  |  |  |  |  |
| 24 | TES | M | 25 | 27.14 | 23 | 0 |  |  |  |  |  |
| 25 | VID | F | 39 | 26.03 | 23 | 30 |  |  |  |  |  |
| 26 | YUN | F | 32 | 26.03 | 31 | 93 |  |  |  |  |  |
